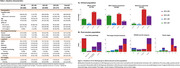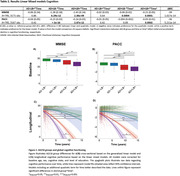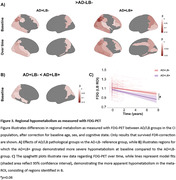# Lewy body pathology exacerbates brain hypometabolism and cognitive decline in Alzheimer’s disease

**DOI:** 10.1002/alz.090655

**Published:** 2025-01-09

**Authors:** Lyduine E. Collij, Sophie E. Mastenbroek, Niklas Mattsson‐Carlgren, Olof Strandberg, Ruben Smith, Sebastian Palmqvist, Rik Ossenkoppele, Oskar Hansson, the Alzheimer's Disease Neuroimaging Initiative

**Affiliations:** ^1^ Amsterdam UMC, Amsterdam Netherlands; ^2^ Lund University, Lund Sweden; ^3^ Amsterdam UMC, location VUmc, Amsterdam Netherlands; ^4^ Clinical Memory Research Unit, Department of Clinical Sciences, Lund University, Lund Sweden; ^5^ Department of Neurology, Skåne University Hospital, Lund Sweden; ^6^ Clinical Memory Research Unit, Department of Clinical Sciences Malmö, Faculty of Medicine, Lund University, Lund Sweden; ^7^ Wallenberg Center for Molecular Medicine, Lund University, Lund Sweden; ^8^ Memory Clinic, Skåne University Hospital, Malmö Sweden; ^9^ Alzheimer Center Amsterdam, Amsterdam UMC, Amsterdam Netherlands

## Abstract

**Background:**

Identification of concomitant Lewy Body (LB) pathology might be important for the diagnostic and prognostic work‐up of Alzheimer’s disease (AD) in clinical practice and trials. Here, we aimed to determine whether the presence of LB pathology exacerbates AD‐related disease progression.

**Methods:**

Cognitively impaired (Mild Cognitive Impairment [MCI] and dementia, n=795) individuals from the ADNI cohort with available CSF α‐synuclein amplification assay (SAA) and CSF p‐tau_181_/Aβ_42_ measures were included. Linear mixed models (LMMs) with random slope and intercept were run to assess group effects on longitudinal cognition and cortical metabolism using FDG‐PET, corrected for age, sex, and baseline cognitive state, and education in case of cognition models. To investigate potential non‐linear changes in cognitive functioning, LMMs additionally included an interaction between AD/LB group and time squared (time^2^). Differences between AD/LB groups (n=61) in pathological scores were assessed using ordinal logistic regression models, corrected for sex, age at death, measurement interval and post‐mortem interval.

**Results:**

Participants were on average 75 years of age (SD=7.89), 40.8% were female, and 184 (23.1%) had no biomarker evidence of AD/LB pathology, 39 (4.9%) had isolated LB pathology (AD‐LB+), 395 (49.7%) had only AD pathology (AD+LB‐), and 177 (22.3%) had both pathologies (AD+LB+, Table 1/Figure 1). At baseline, the AD+LB+ group showed worst performance for most cognitive outcomes compared to the other groups, including the AD+LB‐ group (MMSE: p=0.008; PACC: p<0.0001). Over time, both the AD+LB‐ and AD+LB+ groups deteriorated faster in global cognitive function compared to the AD‐ groups. Importantly, this decline was more pronounced for the AD+LB+ group when compared to the AD+LB‐ group (MMSE: β_AD/LB group*time_^2^=‐0.15, p=0.005, PACC: β_AD/LB group*time_^2^=‐0.026, p=0.0015, Table 2/Figure 2). Furthermore, the AD+LB+ group showed more cortical hypometabolism at baseline and over time compared to the AD+LB‐ group, particularly in posterior brain regions (Figure 3). Neuropathological examination showed that the α‐syn SAA had 96.3% (26/27) sensitivity and 96.4% (27/28) specificity (Figure 1).

**Conclusion:**

In a cognitively impaired AD population, co‐existing LB‐positivity exacerbates cognitive decline and cortical brain hypometabolism. In vivo detection of LB pathology could improve the prognostic work‐up of AD in clinical practice and might have implications for the design of clinical AD trials.